# Deciphering immune-inflammatory dysregulation in the endometriotic microenvironment: insights from single-cell omics and artificial intelligence

**DOI:** 10.3389/fimmu.2026.1848643

**Published:** 2026-06-11

**Authors:** Xiaofeng Zou, Xi Wang, Bin Xiong, Mingyang Wang, Danhe Yang, Zhenjiang Lin, Zhiliang Wang

**Affiliations:** Department of Obstetrics and Gynecology, Affiliated Hospital of Zunyi Medical University, Zunyi, Guizhou, China

**Keywords:** articial intelligence, endometriosis, immune-inflammatory dysregulation, machine learning, precision immunotherapy

## Abstract

Endometriosis is a prevalent chronic inflammatory gynecological disorder affecting approximately 10% of reproductive-age women worldwide, characterized by endometrial-like tissue outside the uterine cavity. Ectopic lesion growth tracks closely with immune-inflammatory dysregulation—altered macrophage polarization, impaired natural killer (NK) cytotoxicity, skewed T cell subsets, B cell–related autoimmunity, tolerogenic dendritic cells, mast cell–associated neuroinflammation, and abnormal cytokine networks. Even after many years of study, several regulatory mechanisms in the endometriotic microenvironment remain only partly defined. Single-cell omics—especially single-cell RNA sequencing (scRNA-seq), spatial transcriptomics, mass cytometry (CyTOF), and multi-omics integration—maps immune composition and cell–cell communication at a level bulk assay typically miss, including rare states and niche structure. Artificial intelligence (AI) and machine learning (ML), including single-cell foundation models, deep learning for drug repurposing, immune deconvolution, and large language models, are now common choices for integrating large datasets, deriving immune signatures, ranking candidate targets, and supporting translation. This review summarizes recent work at that interface: immune heterogeneity and dysfunction across macrophage, NK, T, B, dendritic cell, and mast cell compartments; AI-assisted biomarker studies, repurposing, and network pharmacology, including natural products and traditional Chinese medicine; and practical limits that still affect clinical application.

## 1 Introduction

Endometriosis is a chronic, estrogen-dependent inflammatory disorder defined by the implantation and growth of endometrial-like tissue at extrauterine sites, most commonly the pelvic peritoneum, ovaries, and rectovaginal septum (1). Affecting an estimated 190 million women globally, which accounts for approximately 10% of reproductive-age women, the disease manifests with debilitating pelvic pain, dysmenorrhea, dyspareunia, and infertility, imposing substantial burdens on quality of life and healthcare systems (2). Despite its prevalence, the average diagnostic delay remains 7–10 years, reflecting the absence of reliable non-invasive biomarkers and the continued reliance on laparoscopic visualization for definitive diagnosis (3). This diagnostic gap underscores the urgent need for a deeper mechanistic understanding of disease pathobiology and the development of novel biomarker-driven approaches. To address this unmet clinical demand, it is critical to systematically elucidate the complex molecular and cellular mechanisms underlying endometriosis initiation and progression.

The pathogenesis of endometriosis extends far beyond Sampson’s retrograde menstruation theory. Contemporary understanding positions the disease as a chronic inflammatory condition in which cyclic hemorrhage within ectopic implants triggers a self-perpetuating cascade of inflammation, immune activation, neoangiogenesis, and fibrosis (4). Central to this pathological cycle is the tissue microenvironment surrounding ectopic lesions, a dynamic milieu comprising immune cells (macrophages, natural killer cells, T and B lymphocytes, dendritic cells, and mast cells), endometrial stromal and epithelial cells, fibroblasts, endothelial cells, and a complex network of soluble mediators including cytokines, chemokines, and growth factors (5). Interactions among these cellular and molecular components favor immune evasion, lesion survival, and disease progression, yet many regulatory details remain unclear. Recent conceptualization of endometriosis as an immune-mediated disease has further highlighted the centrality of immune dysregulation in its pathogenesis (6).

Traditional bulk transcriptomic and proteomic studies have provided foundational knowledge regarding immune alterations in endometriosis but are inherently limited by averaging signals across heterogeneous cell populations, thereby obscuring critical cell-type-specific and cell-state-specific information (7, 8). Single-cell omics technologies, such as single-cell RNA sequencing (scRNA-seq), single-cell assay for transposase-accessible chromatin sequencing (scATAC-seq), cytometry by time-of-flight (CyTOF), and spatial transcriptomics, have improved the ability to parse cellular complexity in the endometriotic microenvironment at single-cell and spatial resolution (9, 10). These technologies have enhanced our ability to resolve cellular complexity in the endometriotic microenvironment at single-cell and spatial resolution. These approaches have identified immune subtypes, transitional states, and communication patterns that bulk data often blur.

Artificial intelligence (AI) and machine learning (ML) methods are now standard for analyzing the high-dimensional, multi-layered datasets produced by single-cell workflows and for extracting biologically and clinically useful patterns (11). Together, single-cell omics and AI shift emphasis from description alone toward more predictive and translational questions about the immune-inflammatory microenvironment.

We summarize immune-inflammatory dysregulation in the endometriotic microenvironment, with weight on single-cell omics and AI. Three guiding questions structure the review: which immune-cell states are most consistently linked to lesion persistence and symptom burden; what single-cell and spatial data add beyond bulk profiling for mechanism-level interpretation; and where AI provides incremental value for biomarker stratification and therapeutic nomination under real-world constraints.

## 2 Single-cell omics unraveling the immune landscape of endometriosis

**Figure 1:**
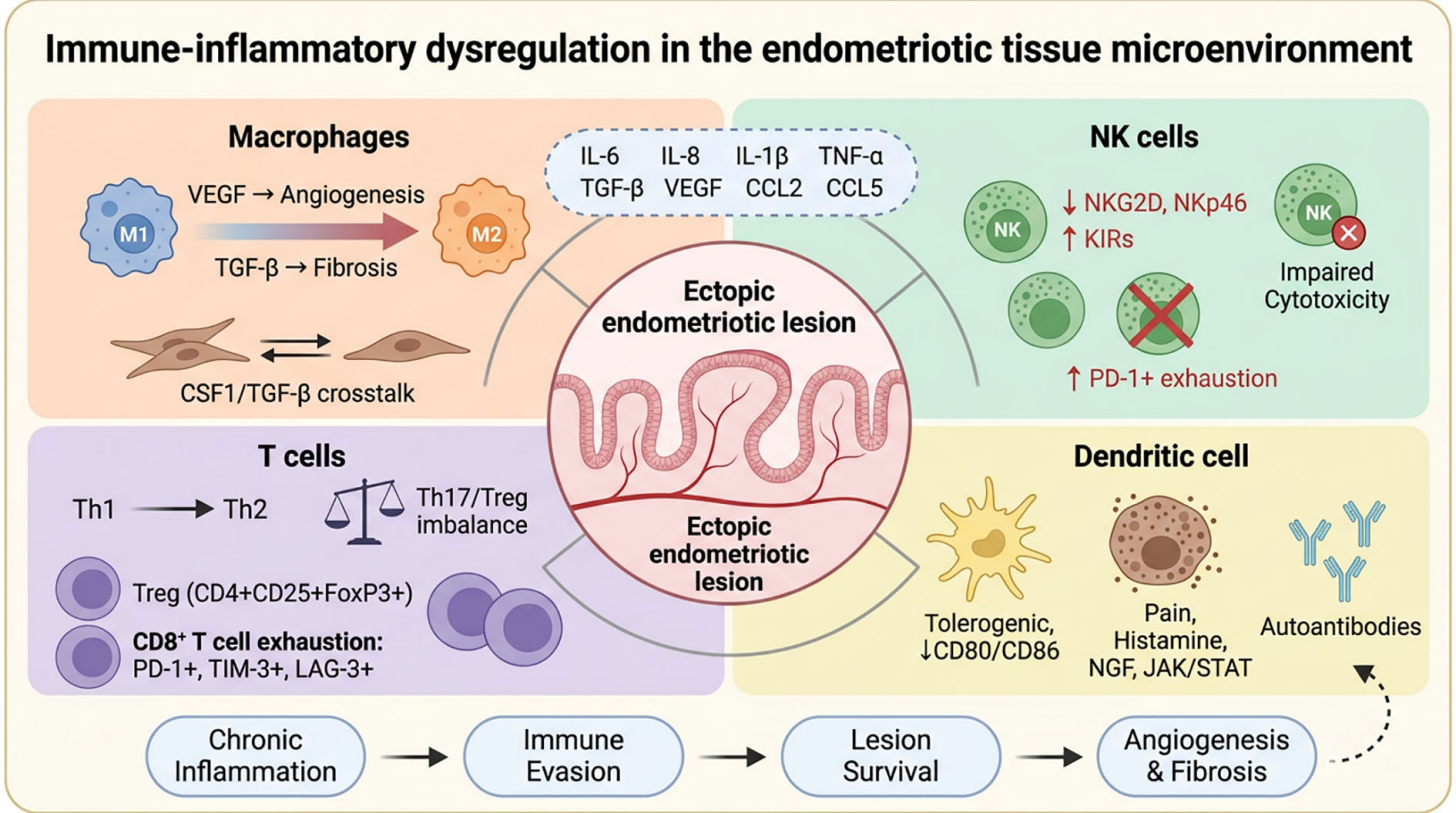
Immune-inflammatory dysregulation in the endometriotic tissue microenvironment. Summary of major immune changes around ectopic lesions. Macrophages shift toward an M2-like program (e.g., IL-4, TGF-β), supporting angiogenesis (VEGF) and fibrosis and exchanging signals with stromal cells (CSF1/TGF-β). NK cells show reduced cytotoxicity (lower NKG2D, NKp46; higher inhibitory KIRs) and PD-1-associated exhaustion in advanced disease. T cells show Th2 skewing, Treg expansion, Th17/Treg imbalance, and exhausted CD8+ cells (PD-1, TIM-3, LAG-3). B cells contribute autoantibodies; dendritic cells skew tolerogenic; mast cells link inflammation to pain (histamine, NGF; JAK/STAT–nerve interactions). Cytokines such as IL-6, IL-8, IL-1β, TNF-α, TGF-β, VEGF, and CCL2 help sustain inflammation and lesion persistence.

**Figure 2:**
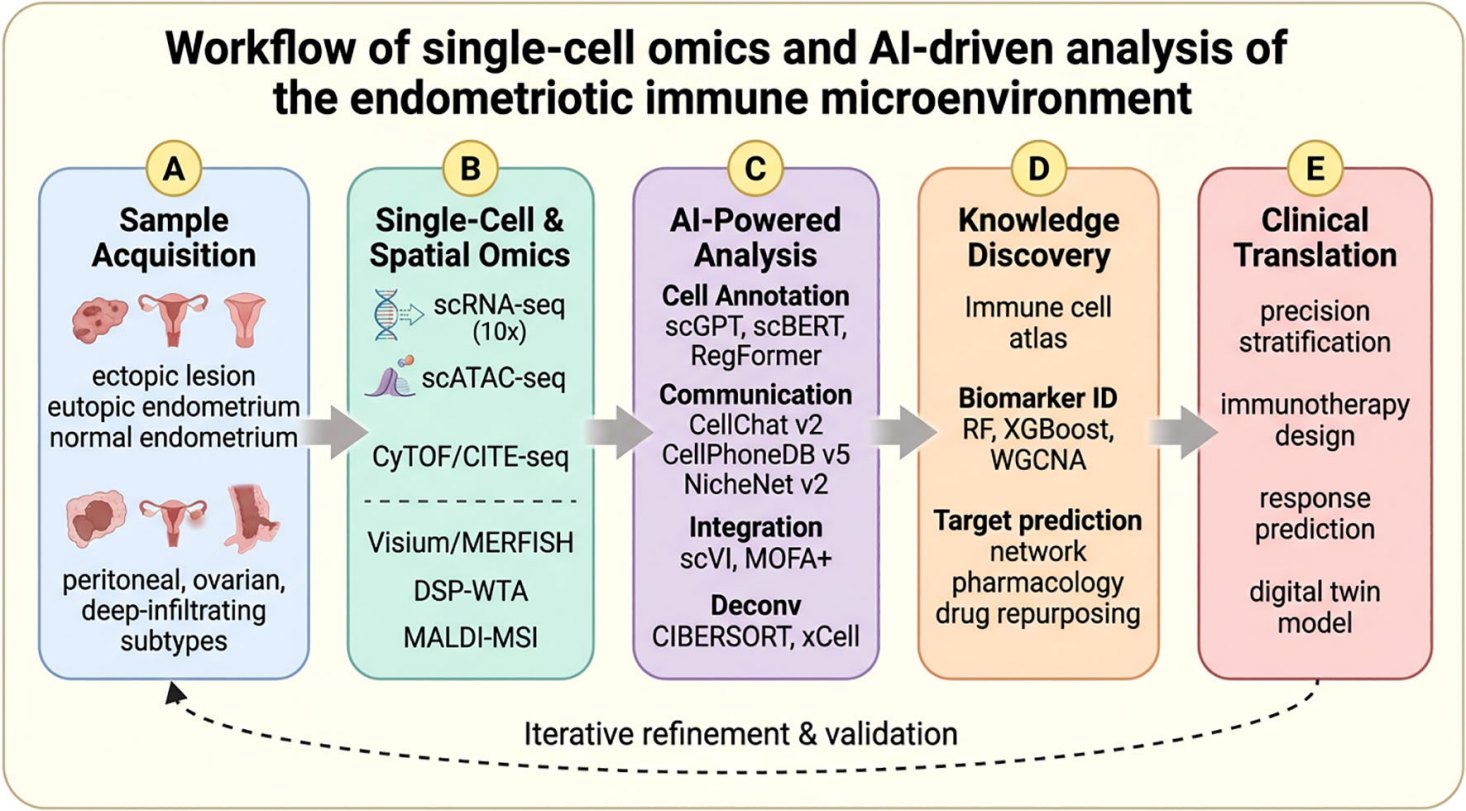
Workflow of single-cell omics and AI-driven analysis of the endometriotic immune microenvironment. Stages from specimen to translation. **(A)** Tissue sources: ectopic, eutopic, and normal endometrium (peritoneal, ovarian, deep-infiltrating). **(B)** Assays: scRNA-seq (e.g., 10x Chromium), scATAC-seq, CyTOF, CITE-seq, spatial platforms (Visium, MERFISH, DSP-WTA, MALDI-MSI). **(C)** Computation: foundation models (scGPT, scBERT, Geneformer, RegFormer), communication tools (CellChat v2, CellPhoneDB v5, NicheNet v2), deconvolution (CIBERSORT, xCell, MCP-counter), integration (scVI, MOFA+, Seurat v5 WNN). **(D)** Outputs: atlases, ML-based signatures (e.g., random forest, XGBoost, WGCNA), target and repurposing hypotheses from network pharmacology or dedicated AI screens. **(E)** Intended downstream uses: risk stratification, trial design, response modeling, and (still largely conceptual) digital twin–style integration.

The endometriotic tissue microenvironment (**Table 1**; **Figure 1**) is characterized by a chronically inflamed milieu in which immune surveillance is subverted and pro-inflammatory signals coexist paradoxically with immunosuppressive mechanisms that facilitate lesion persistence (4, 12). While traditional bulk-level research is limited in resolving cellular heterogeneity, single-cell sequencing enables precise profiling of immune cell subsets and dynamic cellular states, offering novel and in-depth insights into immune dysregulation within endometriotic lesions. **Figure 2** details the holistic AI-driven single-cell omics workflow used to navigate this intricate terrain, illustrating a trajectory from high-resolution data collection using single-cell and spatial technologies to the generation of clinically relevant predictive outputs.

**Table 1:** Immune cell functional dysregulation panorama in the endometriotic microenvironment.

Immune cell	Key functional alterations	Molecular markers/mediators	Single-cell discoveries	Therapeutic potential
Macrophages (M2)	M1→M2 polarization; pro-angiogenic/pro-fibrotic secretion; MMT; impaired phagocytosis	VEGF, TGF-β, MMP9, SPP1, SIRPα, CD64; iron overload	SPP1+ lipid-associated subset; FOLR2+ immunosuppressive subset; differentiation trajectories mapped	CSF1R inhibitors; TGF-β blockade; iron chelation
NK cells	↓Cytotoxicity; ↓activating receptors; ↑inhibitory receptors; metabolic reprogramming; PD-1+ exhaustion	↓NKG2D, NKp46, NKp44; ↑KIRs; GNLY, PRF1	CD56bright CD16− decidual-like subset; exhaustion vs. activation gene modules	Checkpoint modulation; cytokine stimulation; adoptive NK cell transfer
CD8+ T cells	Functional exhaustion; ↓cytolytic capacity; ↓proliferation	PD-1+, TIM-3+, LAG-3+, TOX+; ↓perforin, granzymes	Clonally expanded exhausted subsets; TRM with unique transcriptional profiles	Checkpoint immunotherapy (with caution for benign disease)
CD4+ T cells (Th1/Th2)	Th1→Th2 skewing; ↑IL-4, IL-5, IL-10; ↓IFN-γ, TNF-α	Th2 cytokine signature	Compartmentalized Th2 responses across ectopic/eutopic sites	Cytokine modulation
Tregs	Enrichment in PF and lesions; active immune suppression	CD4+CD25+FoxP3+; IL-10, TGF-β	eTreg vs rTreg heterogeneity; Th17/Treg axis perturbation	Treg depletion strategies
Th17 cells	↑IL-17A; sustained inflammatory signaling; correlated with disease severity	IL-17A, RORγt	Th17/Treg imbalance quantified at single-cell level	IL-17 neutralization
γδ T cells	Dual pro-inflammatory/immunoregulatory roles	Context-dependent cytokine profiles	Subtype-specific roles emerging	Under investigation
B cells	Autoantibody production; Breg dysfunction; IL-24+ regulatory subsets	Anti-endometrial Abs, anti-nuclear Abs; IL-24	Plasma cell enrichment; B cell stimulator overexpression	Autoantibody-based diagnostics
Dendritic cells	Tolerogenic phenotype; ↓antigen presentation	↓CD80, ↓CD86; immature DC markers	pDC vs cDC functional divergence	DC-based vaccination
Mast cells	Neuroinflammation; pain mediation; histamine/NGF release	Histamine, tryptase, NGF; TRPV1/TRPA1; MRGPRX2	Spatial heterogeneity of nerve–mast cell interactions	Mast cell stabilizers; antihistamines; TRPV1 antagonists

scRNA-seq has substantially clarified immune heterogeneity in the endometriotic microenvironment (**Table 2**). Pioneering studies by Fonseca et al. (9) and Tan et al. (10) profiled ectopic, eutopic, and normal endometrial tissues at single-cell resolution, revealing an immune cell composition in ectopic lesions markedly distinct from that of eutopic endometrium. Delving into these architectural differences, comparative analyses have revealed site-specific immune signatures: ectopic lesions are enriched in M2-like macrophages, exhausted T cells, and immunosuppressive Tregs relative to eutopic endometrium. Furthermore, eutopic tissue from endometriosis patients already shows subclinical immune alterations compared to healthy controls, suggesting systemic immune priming. Within this complex immune landscape, Tan et al. analyzed over 122,000 cells across 14 individuals and identified a specific immunotolerant peritoneal niche, notably discovering a perivascular mural cell unique to peritoneal lesions with dual roles in angiogenesis promotion and immune cell trafficking (10). Despite these transformative insights, synthesizing single-cell data across independent cohorts presents challenges. Comparative analysis (**Table 3**) of immune findings across major single-cell studies highlights both consistent themes and notable discrepancies, particularly in macrophage subtype nomenclature and NK cell characterization depth. To overcome these single-study limitations and enhance clinical applicability, integration of scRNA-seq with bulk transcriptomic data has further identified key immune genes and enabled the construction of predictive diagnostic models with high accuracy, successfully bridging single-cell resolution with population-level validation (13).

**Table 2:** Key single-cell omics studies characterizing the immune microenvironment in endometriosis.

Study	Technology	Sample types & size	Key immune findings	Computational methods	Ref
Fonseca et al., 2023	scRNA-seq	370,000+ cells; endometriomas, lesions, eutopic, ovary, peritoneum	SPP1+ macrophages; FOLR2+ immunosuppressive macrophages; ARID1A mutation effects; complement dysregulation; macrophage differentiation trajectories	Seurat, CellChat, NicheNet, RNA velocity, Monocle	(9)
Tan et al., 2022	scRNA-seq + IMC	122,000+ cells; ectopic, eutopic, normal (n=14)	Immunotolerant peritoneal niche; perivascular mural cells; macrophage heterogeneity; T cell exhaustion; tissue-resident memory T cells	Seurat, CellChat, trajectory analysis, imaging mass cytometry	(10)
Zhang et al., 2025	High-resolution spatial transcriptomics	Ovarian and peritoneal lesions	Fibroblast compartments around glands; neuronal and macrophage subset distributions; epithelial–neuronal interactions	Spatial analysis, organoid validation	(13)
Vallvé-Juanico et al., 2022	CyTOF	Endometrium and peripheral blood	CD91+ macrophages overexpressing SIRPα and CD64; MPC system dysregulation	Mass cytometry, FlowSOM	(14)
Burns et al., 2025	Spatial transcriptomics (iScience)	Superficial peritoneal lesions, eutopic endometrium	Epithelium-to-macrophage signaling 3.7× higher in lesions; C3-mediated pathobiology	Spatial deconvolution, CellChat	(15)
Wu et al., 2024	scRNA-seq	Ovarian endometriomas	IGFBP5+ pro-inflammatory macrophages; NK cell exhaustion; aberrant epithelial proliferation	Seurat, GSVA, CellChat	(16)
Liu et al., 2025	scRNA-seq + bulk RNA-seq	Ectopic lesions, integrated datasets	GNLY, PRF1, ENTPD1 as NK cell dysfunction biomarkers; ML-validated immune signatures; GBP2, HCK as ovel therapeutic targets	Machine learning, CIBERSORT, AI target discovery	(17)
Qi et al., 2025	scRNA-seq + spatial (DSP-WTA) + metabolomics	Ovarian endometriomas	XBP1, VCAN, CLDN7 epithelial markers; THBS1 in perivascular cells; altered cholesterol metabolism	Multi-omics integration, MALDI-MSI	(18)

**Table 3:** Cross-study comparison of immune cell findings from single-cell omics studies in endometriosis.

Feature	Tan et al., 2022 (10)	Fonseca et al., 2023 (9)	Zhang et al., 2025 (13)	Vallvé-Juanico et al., 2022 (14)	Burns et al., 2025 (15)	Wu et al., 2024 (16)	Liu et al., 2025 (17)	Qi et al., 2025 (18)
Technology	scRNA-seq + IMC	scRNA-seq	High-resolution spatial transcriptomics	CyTOF	Spatial transcriptomics (iScience)	scRNA-seq	scRNA-seq + bulk RNA-seq	scRNA-seq + spatial (DSP-WTA) + metabolomics
Sample size	122K+ cells, n=14	370K+ cells	Ovarian and peritoneal lesions	Endometrium and peripheral blood	Superficial peritoneal lesions, eutopic endometrium	Ovarian endometriomas	Ectopic lesions, integrated datasets	Ovarian endometriomas
Macrophage subtypes	M2-like enriched	SPP1+, FOLR2+ subsets	Macrophage subset distributions	CD91+ macrophages overexpressing SIRPα and CD64	Epithelium-to-macrophage signaling 3.7× higher in lesions	IGFBP5+ pro-inflammatory macrophages	—	—
NK cell findings	Functional impairment	—	—	—	—	NK cell exhaustion	GNLY, PRF1, ENTPD1 as NK cell dysfunction biomarkers	—
T cell exhaustion	PD-1+, TIM-3+ CD8+	TOX+ clonal expansion	—	—	—	—	—	—
Treg enrichment	Confirmed	Confirmed	—	—	—	—	—	—
Key novel finding	Immunotolerant peritoneal niche; perivascular mural cells	Macrophage differentiation trajectories; ARID1A mutation effects	Fibroblast compartments around glands; epithelial-neuronal interactions	MPC system dysregulation	C3-mediated pathobiology	Aberrant epithelial proliferation	ML-validated immune signatures; GBP2, HCK, ITGB2 targets	XBP1, VCAN epithelial markers; altered cholesterol metabolism
Communication tools	CellChat	CellChat, NicheNet, RNA velocity, Monocle	Spatial analysis	Mass cytometry, FlowSOM	Spatial deconvolution, CellChat	Seurat, GSVA, CellChat	Machine learning, CIBERSORT	Multi-omics integration, MALDI-MSI

### 2.1 Macrophage polarization and dysfunction

Among the diverse immune populations mapped by these single-cell technologies, macrophages have emerged as central drivers of lesion pathology. Macrophages are the most abundant immune cell population in the peritoneal fluid and ectopic lesions of endometriosis patients and strongly shape the local inflammatory milieu (5). Trajectory analysis using RNA velocity and pseudotime algorithms (Monocle, scVelo) has mapped the differentiation trajectories of macrophages from newly recruited monocytes through intermediate inflammatory states to terminally differentiated tissue-resident phenotypes within lesions, providing dynamic insights into immune cell state transitions (9).

Under physiological conditions, peritoneal macrophages efficiently phagocytose retrograde menstrual debris and maintain tissue homeostasis. In endometriosis, however, these cells exhibit a striking shift from a classically activated pro-inflammatory (M1) phenotype toward an alternatively activated anti-inflammatory and pro-fibrotic (M2) phenotype (19). This M1-to-M2 polarization switch is driven by signals within the lesion microenvironment, including IL-4, IL-13, IL-10, and TGF-β, and has profound functional consequences. M2-polarized macrophages promote ectopic lesion survival by secreting pro-angiogenic factors (VEGF), tissue-remodeling enzymes (matrix metalloproteinases), and anti-inflammatory cytokines that suppress effective immune clearance (20). These studies identified previously unrecognized macrophage subpopulations within lesions, including a SPP1+ lipid-associated macrophage subset with enhanced pro-fibrotic and pro-angiogenic transcriptional programs, and a FOLR2+ tissue-resident macrophage population displaying immunosuppressive signatures (9).

Deep immunophenotyping using mass cytometry (CyTOF) has further refined our understanding of macrophage heterogeneity (19). Endometrial CD91+ macrophages in endometriosis patients overexpress SIRPα (a phagocytosis inhibitor) and CD64, suggesting impaired clearance capacity and enhanced inflammatory signaling (14). Macrophages engage in reciprocal crosstalk with endometrial stromal cells through paracrine signaling loops; the interaction between stromal cells and macrophages impairs NK cell cytotoxicity through IL-10 and TGF-β secretion, while stromal cell-derived CSF1 recruits and polarizes macrophages, which in turn promote stromal-to-mesenchymal transition and fibrogenesis (21). Spatial transcriptomic analyses have revealed that epithelium-to-macrophage signaling is 3.7-fold higher in ectopic lesions compared to patient-matched eutopic endometrium, with lesion epithelium orchestrating inflammatory signaling and promoting a pro-repair macrophage phenotype through complement C3-mediated pathways (15). Iron metabolism is also central to macrophage polarization in this setting. Hemoglobin-derived iron from cyclic hemorrhage within ectopic lesions activates peritoneal macrophages, induces oxidative stress, and creates a pro-inflammatory milieu that paradoxically facilitates M2 polarization and lesion survival (22). Ochoa et al. integrated single-cell transcriptomic profiling and artificial intelligence-based computational deconvolution to map endometriosis-related genetic risk loci to myeloid and macrophage lineage cells, and to clarify core immune and inflammatory dysregulation patterns in the endometriotic microenvironment (23). Recent evidence has further established that macrophage-to-myofibroblast transition (MMT) is a key mechanism driving endometriosis-associated fibrosis, with TGFB1/SMAD3 signaling orchestrating the conversion of macrophages into collagen-producing myofibroblasts within ectopic lesions (24, 25). A narrative review has integrated these macrophage-centric themes, linking inflammation, fibrosis, angiogenesis, and immune evasion in the endometriotic microenvironment (26). This self-reinforcing circuit perpetuates chronic inflammation and progressive tissue remodeling.

### 2.2 Natural killer cell functional impairment

Natural killer (NK) cells constitute a critical first-line defense against ectopic endometrial implants through cytolytic activity. In endometriosis, however, peritoneal NK cells display markedly diminished cytotoxic function, which is increasingly recognized as a key factor in the failure of immune surveillance that permits lesion establishment (27).

Dissection of the ovarian endometrioma microenvironment through scRNA-seq identified IGFBP5+ pro-inflammatory macrophages, NK cell exhaustion, and aberrant epithelial proliferation as key pathogenic features (16). Recent investigations have revealed that advanced endometriosis is associated with higher numbers of exhausted PD-1+ NK cells within ectopic lesions, while TIM-3+ NK cell populations are not similarly elevated, suggesting distinct checkpoint-mediated exhaustion pathways in NK cells (28). scRNA-seq-based biomarker analyses have identified granulysin (GNLY), perforin 1 (PRF1), and ENTPD1 as central to NK cell dysfunction in endometriosis; GNLY and PRF1 are predominantly expressed in NK cells and CD8+ T cells and correlate with cytotoxic activation signatures (17). Emerging evidence implicates metabolic reprogramming—including altered autophagy, lipid metabolism, and trogocytosis—in driving NK cell dysfunction, suggesting that the metabolic milieu of ectopic lesions actively suppresses NK cell effector functions beyond receptor-mediated mechanisms (29). NK cell dysfunction may both permit immune escape and fuel disease progression through pro-angiogenic and pro-fibrotic cytokines (30). Another study analyzed single-cell transcriptomic data from the GEO database and identified that PGI2 contributes to endometriosis progression by enhancing the adhesive capacity of endometrial stromal cells and suppressing the cytotoxic activity of natural killer cells (31).

### 2.3 T cell subsets and regulatory imbalance

T lymphocytes in the endometriotic microenvironment exhibit complex alterations across multiple subsets. T cell heterogeneity has been similarly elucidated. Single-cell analyses have identified clonally expanded CD8+ T cells co-expressing exhaustion markers (PDCD1, HAVCR2, LAG3, TOX) within ectopic lesions, alongside tissue-resident memory T cells (TRM) with unique transcriptional profiles, suggesting compartmentalized adaptive immune responses within the endometriotic microenvironment (10).

A consistent finding is the skewing of the T helper cell balance from a Th1-dominant cytotoxic profile toward a Th2-biased response, characterized by reduced secretion of IFN-γ and TNF-α and elevated production of IL-4, IL-5, and IL-10, thereby attenuating cell-mediated immune clearance of ectopic tissue (32, 33). This Th2 polarization creates an anti-inflammatory and tissue-remodeling environment that facilitates lesion persistence within the sterile, non-pathogen-driven inflammatory milieu characteristic of endometriosis.

The Th17/Treg axis is also significantly perturbed. Regulatory T cells (Tregs, CD4+CD25+FoxP3+) are enriched in both the peritoneal fluid and ectopic lesions of endometriosis patients, where they actively suppress effector T cell responses and promote immune tolerance to ectopic implants (34). Conversely, using single-cell transcriptomic analysis, a study reveals that IL-17A–IL-17RA signaling in myeloid cells promotes inflammatory cell recruitment in endometriotic lesions, while gut microbiota–derived chenodeoxycholic acid regulates (35). Th17 cell abundance and represents a potential therapeutic target for endometriosis. The resulting Th17/Treg imbalance perpetuates a state of chronic inflammation coupled with localized immune evasion. Functionally, this axis is context-sensitive: expanded Tregs may dampen damaging inflammation yet simultaneously weaken lesion clearance, whereas Th17-associated signals can sustain inflammatory recruitment and stromal activation.

CD8+ cytotoxic T lymphocytes within ectopic lesions display features of functional exhaustion, including upregulated expression of inhibitory checkpoint molecules PD-1 and TIM-3, diminished production of cytolytic mediators (perforin and granzymes), and impaired proliferative capacity (33, 36). The PD-1/PD-L1 signaling axis has been implicated in peripheral immune regulation in endometriosis pathogenesis, with PD-L1 expression on endometriotic cells contributing to T cell suppression (37). TIM-3 promotes endometriotic cell proliferation, migration, and invasion through the BDNF-mediated PI3K/AKT signaling pathway, suggesting dual roles as both an immune checkpoint and a direct pathogenic mediator (38). Immune checkpoint molecules—including PD-1, PD-L1, TIM-3, CTLA-4, LAG-3, and TIGIT—have emerged as key regulators of the immunosuppressive microenvironment in endometriosis (36). This T cell exhaustion phenotype closely parallels observations in the tumor immune microenvironment, reinforcing the conceptual parallels between endometriosis and cancer immunobiology. Notably, γδ T cells—an unconventional T cell population bridging innate and adaptive immunity—have been identified in endometriotic lesions, where they may serve dual pro-inflammatory and immunoregulatory roles depending on their activation state and subtype composition (39). Together, these observations suggest that T-cell dysregulation in endometriosis spans helper-cell polarization, checkpoint biology, and tissue-resident adaptive memory, rather than a single linear exhaustion pathway.

### 2.4 B cells and autoimmune features

The role of B lymphocytes in endometriosis pathogenesis has gained increasing recognition. B cell activation with autoantibody production represents a key pathogenetic mechanism, supporting the classification of endometriosis as an immune-mediated disease (6, 40). A meta-analysis of 41 studies encompassing 2,825 endometriosis patients and 4,158 controls reported a significant association between serum autoantibodies and endometriosis susceptibility (odds ratio: 4.242), with specific autoantibodies including anti-nuclear antibodies, anti-β2-glycoprotein-1, anti-CA125, anti-carbonic anhydrase 1, anti-cardiolipin, anti-endometrial, anti-laminin-1, and anti-syntaxin antibodies (41). Autoantibodies to tropomyosin have shown diagnostic value with 73.6% sensitivity and 81.5% specificity, while broader autoantibody spectra have been detected in ovarian endometriosis compared to deep infiltrating endometriosis (42).

By performing single-cell RNA sequencing to analyze peritoneal fluid samples, the research team uncovered unique immune cell subtypes in endometriosis patients and confirmed abnormal immune function driving the retention of ectopic endometrial tissue (43). Flow cytometric analysis of peripheral immune characteristics has revealed that immune disorders in endometriosis patients primarily relate to B cell dysfunction and their subsets, alongside abnormalities in CD4+ T cells, CD8+ T cells, and γδ T cells (16). Recent investigations have uncovered that IL-24-producing regulatory B and T lymphocytes are present in endometriotic tissue, suggesting a previously unappreciated immunoregulatory axis involving Breg-like populations that may contribute to local immune tolerance (44). Beyond systemic autoantibody signatures, local B-cell/plasma-cell activity within lesions likely interacts with stromal and myeloid compartments through cytokines and antigen-presentation loops, supporting both chronic inflammation and immune escape in a site-specific manner.

### 2.5 Dendritic cells, mast cells, and neuroinflammation

Dendritic cells (DCs) in ectopic lesions exhibit an immature tolerogenic phenotype with reduced expression of co-stimulatory molecules (CD80, CD86) and impaired antigen-presenting function, further dampening adaptive immune responses and contributing to the immunosuppressive microenvironment (45).

Estrogen mediates the inflammatory role of mast cells in endometriosis pathophysiology, contributing to the disease’s hormone-dependent nature (46). Neuroinflammation is a major driver of chronic pain in endometriosis; the JAK/STAT pathway acts as a shared node for immune activation and nerve fiber growth in ectopic lesions (47). Nerve bundle density and expression of NGF and IL-1β display heterogeneous patterns within endometriotic lesions, varying across disease subtypes and suggesting spatial compartmentalization of neuroimmune interactions. Sensory nerves innervating endometriotic lesions drive chronic pain while also contributing to lesion growth by secreting neurotrophic factors and interacting with immune cells, creating a bidirectional neuroimmune axis. At the molecular level, inflammation-mediated macrophage polarization has been shown to induce TRPV1/TRPA1 heteromers in endometriotic tissue, directly linking immune activation to nociceptor sensitization and pain generation (46). MRGPRX2-mediated mast cell activation sensitizes sensory neurons through the histamine/HRH1/TRPV1 signaling pathway, consistent with preclinical rationale for mast cell–directed analgesic strategies (48).

### 2.6 Cytokine and chemokine networks

Beyond direct cell-to-cell interactions, the coordinated action of these diverse immune cells is fundamentally mediated through a complex, highly dynamic cytokine and chemokine network. The coordinated action of these immune cells is mediated through a complex cytokine and chemokine network. Elevated levels of IL-6, IL-8 (CXCL8), IL-1β, TNF-α, MCP-1 (CCL2), and RANTES (CCL5) in the peritoneal fluid establish a pro-inflammatory chemotactic gradient that recruits and activates immune cells while simultaneously promoting angiogenesis and lesion growth (49). TGF-β serves as a master regulator within this network, driving fibrosis, Treg induction, M2 macrophage polarization, and NK cell suppression across multiple cell types. VEGF, produced by macrophages, stromal cells, and ectopic endometrial tissue, sustains the neovascularization essential for lesion survival (50). Importantly, these mediators form reinforcing loops rather than isolated pathways: recruited myeloid and stromal cells release additional chemokines/cytokines, which amplify immune-cell influx, stromal activation, nociceptive sensitization, and matrix remodeling. This network logic helps explain why inflammatory signaling, lesion persistence, fibrosis, and pain often co-evolve over time. Extracellular vesicles (EVs), including exosomes and microvesicles, have emerged as critical mediators of intercellular communication within this network, transferring immunomodulatory proteins, microRNAs, and lipids between immune cells, stromal cells, and ectopic endometrial tissue to amplify inflammatory and pro-angiogenic signaling cascades (26). The complement system represents another layer of immune dysregulation, with complement components C3 and C5a elevated in peritoneal fluid and ectopic lesions, generating inflammatory mediators that recruit and activate immune cells while promoting lesion vascularization and fibrosis (51). Recent network meta-analysis has further revealed cross-talk between complement and coagulation cascades in endometriosis, identifying JAK inhibitors as potential therapeutic agents targeting this convergence point (52).

### 2.7 Interplay between pathogenic immune imbalance and reparative remodeling

Distinguishing pathogenic immune imbalance from adaptive/reparative remodeling is crucial for understanding endometriosis yet challenging with traditional bulk assays. High-resolution single-cell omics allows us to decouple these intricate processes. For instance, scRNA-seq reveals distinct subclusters of macrophages and fibroblasts within the same lesion. Specific populations are engaged in adaptive/reparative remodeling by clearing ectopic debris and initiating physiological wound healing. In contrast, other subsets drive a pathogenic immune imbalance through perpetuating chronic neuroinflammation and pathological fibrosis (53). By mapping these specific cell states and their developmental trajectories, single-cell technologies provide a precise lens to identify the tipping point where physiological repair fails and pathogenic dysregulation begins (54).

### 2.8 Cell–cell communication networks

scRNA-seq data are widely used to infer intercellular communication networks in the endometriotic microenvironment. Tools such as CellChat (55), CellPhoneDB (56), and NicheNet (57) leverage curated databases of ligand–receptor interactions to predict signaling pathways between cell populations from transcriptomic data. These tools have undergone significant recent updates: CellChat v2 now quantifies signaling communication probability using a mass-action-based model incorporating multisubunit structures and cofactor modulation, with an expanded ligand–receptor database and interactive visualization capabilities (58). CellPhoneDB v5 has expanded its interaction repository by one-third to include non-protein ligands such as endocrine hormones and GPCR ligands, with new spatial-aware methods for prioritizing cell–cell interactions (59). NicheNet v2 provides sender-agnostic and sender-focused analytical approaches for inferring active ligands from transcriptomics data (60).

Applied to endometriosis datasets, these analyses have revealed extensive communication networks between immune and stromal compartments. Key signaling axes identified include the CSF1–CSF1R pathway mediating macrophage recruitment and polarization by stromal cells, the CXCL12–CXCR4 axis facilitating immune cell chemotaxis and lesion vascularization, the SPP1 (osteopontin) signaling network linking macrophages to fibroblast activation and extracellular matrix remodeling, and the TGF-β signaling cascade coordinating immunosuppression across multiple cell types (9, 10). Furthermore, recent studies have broadened our understanding of these networks to include neuroimmune and mesothelial–stromal interactions. For instance, nociceptor-to-macrophage communication via the CGRP/RAMP1 signaling axis has been shown to drive endometriosis-associated pain and lesion growth by shifting macrophages to a pro-endometriosis phenotype (61). Additionally, single-cell analyses have identified critical interactions between endometriosis-associated mesothelial cells (EAMCs) and ectopic stromal cells, demonstrating that intercellular communication mediated by the FN1–AKT pathway may influence progesterone resistance across different subtypes of the disease (62). Recent studies have expanded the scope of single-cell profiling across different endometriosis subtypes. A multi-site scRNA-seq analysis across peritoneal, deep-infiltrating, and ovarian endometriosis identified 44 subpopulations and revealed distinct epithelial–mesenchymal transition processes among subtypes, proposing that endometriosis-associated mesothelial cells influence progesterone resistance in stromal cells through the FN1–AKT signaling pathway (47).

Beyond conventional ligand–receptor analyses, tensor decomposition approaches such as Tensor-cell2cell v2 now enable the simultaneous factorization of protein- and metabolite-mediated cell–cell communication, uncovering coordinated signaling programs that are missed by pairwise interaction methods (63). Complementarily, MEBOCOST maps metabolite-mediated intercellular communication from single-cell transcriptomics data, revealing metabolic signaling axes—including lactate, prostaglandin, and amino acid transport—that may modulate immune cell function within the peritoneal microenvironment (18).

## 3 Spatial transcriptomics and multi-omics integration

scRNA-seq loses spatial information when tissue is dissociated, but it offers deep transcriptional coverage. Spatial transcriptomics (ST) is increasingly used in endometriosis to recover *in situ* context, as exemplified by large-scale spatial transcriptomic atlases of the human endometrium and endometriotic lesions that map cellular niches across menstrual cycle phases (64). As the available modalities expand, systematic benchmarking of imaging ST platforms, including MERFISH, Xenium, CosMx, and Stereo-seq, in FFPE tissues has established critical performance metrics for sensitivity, specificity, and spatial resolution, guiding platform selection for endometriosis tissue analysis (26). Spatial transcriptomics datasets of the human endometrium from clinical conditions such as repeated implantation failure are providing reference atlases that contextualize immune niche organization in both healthy and pathological states (65).

High-resolution spatial transcriptomics has mapped ovarian and peritoneal lesions, identifying shared spatial features including immune cell infiltration zones, fibroblast compartments surrounding epithelial glands, and distinct distributions of neuronal and macrophage subsets (13). Beyond marker localization, these studies indicate spatially constrained immune niches, non-random immune–stromal proximity patterns, and lesion-specific signaling territories that are not inferable from dissociated profiles alone. For instance, ST analysis of superficial peritoneal endometriotic lesions demonstrated that epithelium-to-macrophage signaling is markedly elevated in lesions, identifying complement C3 as having a new role in lesion pathobiology (15). Crucially, these spatial maps serve as a bridge to functional validation, as demonstrated by studies using 3D organoid co-culture models to validate ST-inferred epithelial–neuronal interactions (13).

Complementary single-cell and spatial technologies have further enriched the characterization of the endometriotic microenvironment beyond the transcriptome. Single-cell proteomic technologies, such as CyTOF, have enabled high-dimensional phenotyping of peritoneal immune populations at the protein level, identifying more than 40 distinct immune cell types and revealing dynamic immune signatures correlating with disease severity (14, 66). Similarly, combining scRNA-seq with Digital Spatial Profiler and Matrix-Assisted Laser Desorption/Ionization Mass Spectrometry (MALDI-MS) Imaging in ovarian endometriomas has provided a multi-omic spatial view. This approach identified XBP1, VCAN, and CLDN7 as key epithelial markers and THBS1 in perivascular cells, while concurrently mapping altered cytochrome P450 enzymes and cholesterol metabolism pathways in mesenchymal regions (65).

Integrating these transcriptomic, proteomic, epigenomic, spatial, and metabolomic layers using tools such as MOFA+ and Seurat v5 WNN is one practical route to generating richer maps of the endometriotic immune landscape. However, integration quality remains sensitive to tissue source, menstrual phase, disease stage, dissociation protocol, and platform-specific noise; normalization and harmonization choices can materially change inferred cell states and communication rankings. Therefore, next-step priorities include the joint modeling of cell proximity, ligand–receptor activity, and protein-level readouts through integration with multiplex immunohistochemistry, proteomics, and perturbation assays that test candidate signaling axes in functional systems.

## 4 Artificial intelligence in deciphering the endometriotic immune microenvironment

It is important to clarify the incremental value of AI over conventional bioinformatics workflows when studying the endometriotic immune microenvironment. Traditional pipelines typically rely on linear statistical models that simply catalogue immune cell proportions or identify basic inflammatory genes. While essential, they often miss subtle interactions. In contrast, AI models excel at decoding complex, non-linear immune networks. For instance, AI algorithms can accurately map the dynamic trajectories of macrophages as they transition from reparative to pathogenic states. Furthermore, AI can quantify intricate immune-stromal cross-talk and predict specific immune-modulating drug targets without human threshold biases. Ultimately, AI advances the field from merely describing immune populations to actively deciphering pathological immune mechanisms.

### 4.1 Foundation models and deep learning for single-cell analysis

**Figure 3:**
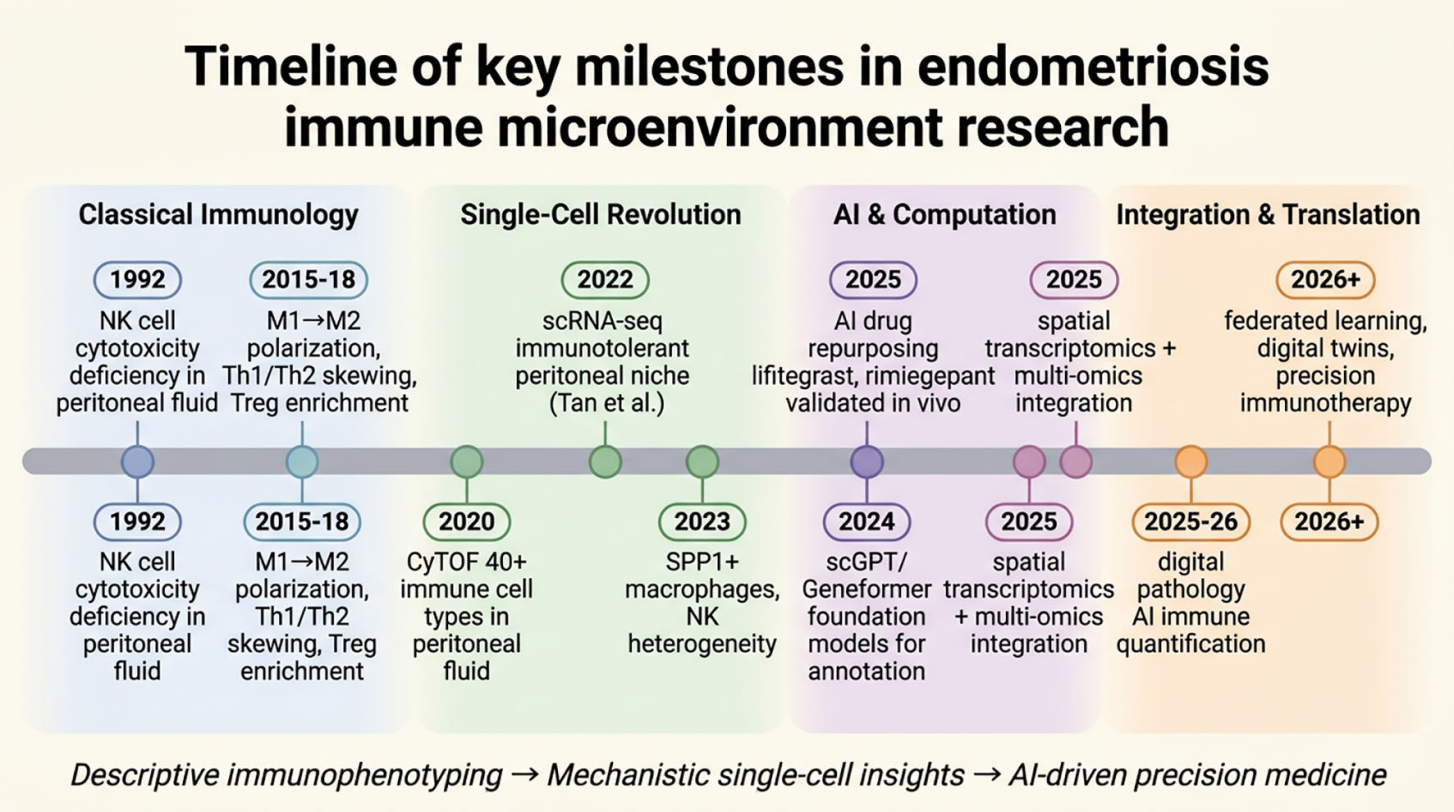
Timeline of key milestones in endometriosis immune microenvironment research. Selected anchors: peritoneal NK dysfunction (1992); M1-to-M2 macrophage polarization; first lesion scRNA-seq maps and immunotolerant niches (2022–2023); CyTOF and spatial mapping; single-cell foundation models for annotation (scGPT, Geneformer, RegFormer, 2023–2025); repurposing candidates with experimental support (lifitegrast, rimegepant, fenoprofen); digital pathology for immune quantification in sectioned tissue (2024–2026). The figure is illustrative, not exhaustive.

Due to the large and heterogeneous nature of single-cell omics datasets, their analysis typically relies on specialized computational pipelines (**Table 4**). To advance these analyses, foundation models for single-cell biology, such as scGPT (67), scBERT (68), and Geneformer (69)have been pre-trained on millions of single-cell transcriptomes (**Figure 3**). These models can then be fine-tuned for automated cell type annotation, gene network inference, and perturbation prediction. For example, scGPT is a generative pretrained transformer trained on over 33 million cells that has demonstrated strong performance in selected annotation tasks and improved rare-state detection in specific benchmark settings (67). Rather than relying on manual curation, these deep learning frameworks enable automated cell type annotation, robust gene regulatory network inference, and in silico perturbation prediction. By leveraging these advanced models, researchers can accurately uncover rare cell states and complex transcriptional programs that traditional computational pipelines might miss, thereby accelerating the discovery of novel cellular phenotypes across diverse biological systems.

**Table 4:** AI and machine learning approaches for analysis of the endometriotic immune microenvironment.

Method category	Representative tools	Application in endometriosis	Key outputs/findings
Foundation Models	scGPT	Cell type annotation; gene network inference; perturbation prediction	Automated identification of rare immune cell states; 33M+ cell pretraining
	scBERT	Cell type annotation from scRNA-seq	Large-scale pretrained deep language model for cell classification
	Geneformer	Transfer learning for network biology	Predictions of gene function and disease association
	RegFormer	Cell annotation; GRN construction; drug response prediction	Outperforms scGPT/Geneformer across multiple tasks; 22M cell pretraining
	scKGBERT	Gene annotation; drug response; disease prediction	Integrates 41M scRNA-seq profiles with 8.9M PPI interactions
Generative Models	scVI (VAE)	Batch correction; data integration; probabilistic modeling	Harmonization of multi-lab endometriosis datasets
	scGen (GAN)	In silico perturbation modeling	Prediction of immune cell responses to stimuli
Cell Communication	CellChat v2	Ligand–receptor interaction inference	CSF1–CSF1R, CXCL12–CXCR4, SPP1, TGF-β signaling in endometriosis
	CellPhoneDB v5	Multi-subunit ligand–receptor analysis	Extended to non-protein ligands; spatial-aware prioritization
	NicheNet v2	Ligand-to-target gene inference	Upstream regulator identification; hierarchical regulatory networks
	GNN-based models	Probabilistic cell–cell communication inference	Signaling pathway activity prediction
Immune Deconvolution	CIBERSORT	Immune cell proportion estimation from bulk RNA-seq	Elevated M2 macrophages and Tregs; reduced NK and CD8+ T cells in ectopic tissue
	xCell	Digital tissue cellular heterogeneity	Comprehensive immune landscape profiling of endometriosis cohorts
	MCP-counter	Tissue-infiltrating immune cell quantification	Population-level immune signature validation
Supervised ML	Random Forest, XGBoost, LASSO, SVM	Diagnostic signature identification; patient stratification	AUC > 0.85–0.95 for non-invasive diagnosis; sMICB and pain score as key features
	WGCNA	Hub gene and gene module identification	CHMP4C, KAT2B, CXCL12, ROBO3, SCG2 as immune hub genes
Network Pharmacology	DrugBank, STRING, DisGeNET integration	Pharmacological landscape mapping; multi-target mechanism analysis	TNF-α, IL-6, JAK-STAT, Notch as druggable nodes; TCM multi-target validation
Drug Repurposing	AI target discovery platforms	Novel target identification and drug repurposing	GBP2, HCK as new targets; lifitegrast, rimegepant, fenoprofen validated
LLMs	GPT-based models, digital twins	Literature mining; hypothesis generation; precision medicine modeling	Digital twin models for gynecological precision medicine

More recent models have expanded this landscape: RegFormer, pretrained on 22 million human cells with approximately 50 million parameters, outperforms scGPT, Geneformer, and scBERT across several reported annotation/GRN/drug-response benchmarks (70). scKGBERT integrates 41 million single-cell RNA-seq profiles with 8.9 million protein–protein interactions and reports gains in gene annotation and disease prediction tasks (26). However, rigorous zero-shot evaluation has revealed important limitations of these foundation models, demonstrating that in some settings, scGPT and Geneformer may be outperformed by simpler established methods such as scVI and Harmony for tasks including cell type clustering and batch integration (44). Biology-driven assessments similarly indicate substantial task- and dataset-dependence (71).

In endometriosis specifically, where disease-focused labeled datasets remain relatively small, concerns about training bias, interpretability, and reproducibility across centers remain central. Variational autoencoders (VAEs) and generative adversarial networks (GANs), implemented in tools such as scVI (72) and scGen (73), address critical computational challenges in multi-sample single-cell studies, including batch effect correction, data integration across platforms, and in silico perturbation modeling. These approaches have enabled the harmonization of endometriosis scRNA-seq datasets generated across different laboratories, facilitating meta-analyses with increased statistical power for detecting immune dysregulation signatures. This AI-driven integration facilitates robust meta-analyses with enhanced statistical power to detect subtle immune dysregulation signatures. Consequently, these approaches have empowered the identification of specific macrophage polarization states and exhausted T cell subsets within the peritoneal niche. Furthermore, Graph Neural Networks (GNNs) and attention-based models are now being applied to map probabilistic cell-to-cell communication networks (74). These tools provide a systems-level understanding of how ectopic stromal cells orchestrate local immunosuppression and interact with surrounding immune populations, ultimately aiding in the identification of new therapeutic targets.

### 4.2 Machine learning for immune signature discovery

Beyond single-cell analysis, ML approaches have been extensively applied to bulk transcriptomic and clinical datasets to characterize the immune landscape of endometriosis. Immune cell deconvolution algorithms, such as CIBERSORT (75), xCell (76), and MCP-counter (77),estimate the proportions of immune cell populations from bulk RNA-seq data. Applied to endometriosis cohorts, these methods have consistently identified elevated M2 macrophage and Treg signatures and diminished NK cell and CD8+ T cell signatures in ectopic tissues compared to controls, corroborating single-cell findings at the population level (78).

Supervised ML algorithms, including random forests (79), support vector machine (80), XGBoost (81), and LASSO regression (82), have been employed to identify immune-related gene signatures that discriminate endometriosis patients from healthy controls or stratify disease subtypes and severity. Weighted gene co-expression network analysis (WGCNA) has identified immune-related gene modules correlated with clinical phenotypes, revealing hub genes such as CHMP4C and KAT2B with validated differential expression in ectopic tissues, as well as CXCL12, ROBO3, and SCG2 as core immune genes with high diagnostic value (83). Efferocytosis-related gene signatures have been used to construct diagnostic prediction models integrating immune infiltration analysis (84), while molecular subtyping based on immune infiltration patterns has revealed distinct endometriosis phenotypes with different immunological profiles (85).

Ensemble learning approaches integrating multiple data modalities have achieved promising diagnostic accuracy. An XGBoost model using soluble immune markers (sMICA, sMICB, and immune checkpoint molecules) in blood serum and peritoneal fluid achieved 94% accuracy, 0.95 AUC, and 0.96 F1-score for minimally invasive endometriosis diagnosis, with sMICB serum level and pain score as key predictive features (86). Another ML model integrating inflammatory biomarkers and demographic variables from population-level survey data reported strong classification performance for endometriosis risk prediction (13). Where performance metrics are reported, interpretation should account for validation setting (internal cross-validation vs external cohort testing), cohort size/composition, and potential feature leakage across reused public datasets. A systematic review and meta-analysis of diagnostic accuracy across ML methods for endometriosis has revealed heterogeneous performance, with pooled sensitivities of 81.7–96.7% and specificities of 70.7–91.6% depending on input data type, highlighting the need for standardized evaluation frameworks (80, 87).

### 4.3 Large language models and clinical decision support

Large language models (LLMs) are a newer computational strand in endometriosis research, with uses in literature mining, hypothesis drafting, and multi-source synthesis. LLM-based tools can help scan large publication sets for gaps, suggest mechanistic angles on immune-inflammatory regulation, and rank targets for follow-up experiments (88). LLM-enabled digital twins for precision medicine have been reported in rare gynecological tumors; analogous modeling of patient-specific immune niches in endometriosis remains mostly prospective (89). Combining LLMs with multi-omics pipelines may shorten parts of the discovery-to-translation path, pending rigorous validation.

A clinically useful endometriosis immune digital twin would ideally integrate, at minimum, a patient’s spatial transcriptomic or multiplex imaging profile of ectopic and peritoneal niches, longitudinal immune biomarker trajectories (such as cytokines, soluble checkpoints, and immune-cell proportions from serial sampling), and hormonal or surgical treatment history together with symptom phenotypes. These multimodal data streams could then be analyzed to simulate niche responses to immunomodulatory or hormonal interventions and to help design biomarker-stratified clinical trials, while ensuring patient privacy and prospective validation.

### 4.4 Digital pathology and image-based AI

AI-powered digital pathology represents an emerging complementary approach to molecular profiling for characterizing the endometriotic immune microenvironment. Deep learning-based tissue segmentation and cell identification algorithms applied to multiplex-stained histological sections have demonstrated the ability to automatically identify and quantify immune cell populations within endometriotic lesions with accuracy comparable to expert pathologists (90). A systematic meta-analysis of diagnostic ML accuracy for endometriosis across imaging and histopathological modalities has confirmed the potential of AI-assisted pathological assessment while identifying substantial heterogeneity in study design and validation approaches (87). Joint use of digital pathology and spatial transcriptomics could improve spatial immune phenotyping of endometriotic lesions by linking morphology to molecular readouts at tissue scale.

## 5 Therapeutic implications

Although current clinical management of endometriosis still relies predominantly on hormonal suppression and surgical intervention (2), the emergence of single-cell atlases and AI-derived targets provides a crucial foundation for novel immune-directed therapies. Specifically,recent scRNA-seq profiling of ovarian endometriosis, such as studies comparing tissues before and after GnRHa treatment, has revealed distinct cell subtype responses to hormonal therapy (87). These dynamic insights into treatment-induced immune remodeling provide a critical data foundation for AI-based pipelines, which are now widely used to nominate therapeutic targets and repurpose existing drugs. Computationally, network pharmacology (91) approaches integrate drug–target interaction databases (such as DrugBank and STITCH) (92), protein–protein interaction networks (such as STRING), and disease–gene association databases (such as DisGeNET) (93) to systematically map the pharmacological landscape of endometriosis. These methods have identified both established inflammatory nodes, including TNF-α, IL-6, and COX-2, and novel druggable pathways, such as JAK-STAT, Notch, and Hippo signaling. To capture even more complex multi-relational drug–disease–target structures that simpler network summaries might omit, advanced knowledge graph embedding methods are increasingly employed to learn low-dimensional representations from vast biomedical networks.

These robust computational predictions have yielded several experimentally validated therapeutic strategies. For instance, an AI-driven platform identified guanylate-binding protein 2 (GBP2) and hematopoietic cell kinase (HCK) as previously unreported therapeutic targets, while also highlighting integrin beta 2 (ITGB2) as a prime candidate for drug repurposing (17). Experimental validation confirmed that all three proteins are upregulated in human endometriotic specimens. Subsequent siRNA knockdown of GBP2 and HCK reduced cell viability and stimulated apoptosis, while lifitegrast, an approved ITGB2 antagonist, effectively suppressed lesion growth in both subcutaneous and intraperitoneal mouse models. Similarly, an integrated multimodal approach identified receptor tyrosine kinase-like orphan receptor 1 (ROR1) as an upregulated target in endometriosis. This specific pipeline predicted rimegepant, a clinically approved CGRP antagonist, as a potential therapy, which later demonstrated concentration-dependent antiproliferative effects in patient-derived organoid models (94). Furthermore, a separate computational transcriptomics-based pipeline identified fenoprofen as a top therapeutic candidate capable of successfully alleviating endometriosis-associated pain in animal models.

Despite these promising discoveries, current AI-driven analyses are best treated as hypothesis-generating tools. Network topology alone cannot resolve critical pharmacological factors such as edge confidence, pharmacokinetics, and dose-response behavior (95). Across all current examples, the supporting evidence remains strictly preclinical, relying on cell, organoid, or animal models. Consequently, these computational predictions must be interpreted as prioritization signals that require rigorous prospective human validation before entering clinical practice.

### 5.1 Precision immunotherapeutic strategies

Recent single-cell and multi-omic data have highlighted several promising directions for immunomodulatory intervention. For instance, the discovery of exhausted CD8+ T cells expressing PD-1 and TIM-3 in ectopic lesions introduces the potential for immune checkpoint modulation. However, deploying such therapies requires careful consideration, as the immunological context of endometriosis fundamentally differs from that of malignancies (33, 36). Additionally, targeting the M2 macrophage polarization axis through CSF1R inhibition or TGF-β pathway blockade has emerged as a highly promising strategy. This approach is directly supported by recent single-cell characterizations of macrophage heterogeneity within the local microenvironment (19). Furthermore, because the JAK/STAT pathway serves as a critical convergence point for both immune activation and neuroinflammation, JAK inhibitors present compelling dual-action candidates capable of simultaneously targeting lesion progression and pain generation (47).

Despite their immense therapeutic potential, these avenues currently remain exploratory strategies grounded in mechanistic insights rather than immediate clinical standards. Moving forward, AI-driven precision stratification based on individual immune microenvironment profiles will be essential to enable tailored therapeutic selection, thereby replacing the conventional universal treatment paradigm.

### 5.2 Natural products and traditional Chinese medicine

Natural products and traditional Chinese medicine (TCM) formulations have garnered increasing attention as modulators of the endometriotic immune microenvironment. Compounds including curcumin, resveratrol, berberine, and ginsenosides have demonstrated immunomodulatory effects in preclinical models—suppressing M2 macrophage polarization, restoring NK cell cytotoxicity, and rebalancing Th17/Treg ratios. Network pharmacology and AI-based analyses have systematically identified the multi-target mechanisms of these natural compounds, revealing their capacity to modulate multiple nodes of the immune-inflammatory network simultaneously. Specific TCM formulations have undergone rigorous network pharmacology analysis: Luoshi Neiyi prescription identified 34 absorbed blood components with core targets including IL-6, EGFR, HIF1A, and EZH2 (96); Wen Jing Tang demonstrated effects through validated immune-inflammatory pathways (97); and Sanjie Zhentong Capsule identified 28 active components targeting 52 therapeutic endpoints involving oxidative stress, steroid metabolism, apoptosis, and proliferation regulation (84). This multi-target pharmacological profile fits the multifactorial immune disturbance seen in endometriosis and is mainly supported by preclinical and network-level studies to date. Network pharmacology should therefore be interpreted as a prioritization framework that requires pharmacokinetic characterization, dose standardization, batch-quality control, and prospective clinical testing before therapeutic claims can be advanced.

To date, few studies have used single-cell RNA sequencing or spatial profiling to directly examine how these natural products or TCM formulations influence specific immune cell populations within endometriotic lesions. A priority for future research is to integrate perturbation experiments with single-cell or spatial readouts and assess changes in immune states, thereby aligning natural-product studies more closely with the single-cell research framework.

## 6 Discussion

### 6.1 Challenges and limitations

Method development has moved quickly, but several barriers still separate most findings from routine clinical use. Current single-cell datasets are limited by relatively small sample sizes, cross-sectional study designs that cannot capture longitudinal immune dynamics, and underrepresentation of diverse populations and endometriosis subtypes. The standardization of computational pipelines and the establishment of shared data repositories and reference atlases are essential for ensuring reproducibility and facilitating collaborative meta-analyses. While spatial transcriptomics has begun to address the loss of spatial context inherent in dissociative scRNA-seq, current platforms still face trade-offs between spatial resolution and transcriptomic depth.

AI models require rigorous external validation in independent clinical cohorts before clinical deployment. Issues of model interpretability and biological plausibility must be carefully addressed—particularly for deep learning approaches where the “black box” nature may hinder mechanistic understanding and clinical acceptance (11). Many of the ML-derived diagnostic signatures and hub genes identified across studies show limited overlap, raising concerns about generalizability and the influence of cohort-specific confounders. The gap between computational prediction and experimental/clinical validation remains substantial, necessitating systematic translational pipelines that integrate in silico discovery with *in vitro*, *in vivo*, and ultimately clinical validation.

Beyond statistical and biological limitations, deploying large foundation models for single-cell analysis (e.g., scGPT, scBERT, RegFormer) imposes substantial computational barriers, including high GPU memory requirements, long training or fine-tuning times, and non-trivial hardware and cloud costs. These resource demands can limit routine adoption in hospital or mid-size academic settings and reinforce the practical value of lighter, interpretable baselines (e.g., scVI/Harmony) for many endometriosis workflows.

A critical technical challenge lies in the standardization and quality control of single-cell datasets used for meta-analysis. Batch effects arising from differences in sample processing, sequencing platforms, and computational pipelines can confound biological signals; recent systematic evaluation has demonstrated that many widely used batch correction methods are poorly calibrated, with Harmony emerging as the only method that consistently performs well across diverse testing scenarios (98). Tissue dissociation protocols also enrich for cells that survive digestion, which can skew inferred immune proportions—an issue when comparing infiltration across subtypes and sites. Additional heterogeneity from disease stage, hormonal treatment status, tissue source (peritoneal/ovarian/deep lesions), and menstrual-cycle context further complicates cross-cohort harmonization and must be modeled explicitly during normalization and integration.

The parallels between endometriotic and tumor immune microenvironments have raised the possibility of immune checkpoint modulation as a therapeutic strategy. While preclinical evidence supports the involvement of PD-1/PD-L1, TIM-3, and other checkpoint molecules in endometriosis pathogenesis, the application of checkpoint inhibitor therapy to a benign, chronic disease in reproductive-age women raises substantial safety and ethical concerns given the autoimmune adverse events associated with these agents (85). Cross-disease comparisons have revealed that the endometriotic immune microenvironment displays a dominant M2 macrophage profile with elevated iron and oxidative stress, fundamentally diverging from the M1-predominant, pro-inflammatory tumor microenvironment of high-grade serous ovarian cancer, cautioning against direct extrapolation of cancer immunotherapy paradigms to endometriosis (99, 100).

### 6.2 Future directions

Near-term priorities include larger, multi-center single-cell and spatial atlases that span subtypes, stages, and ancestries; longitudinal sampling to track immune remodeling during progression and therapy; and trial designs that stratify patients using reproducible immune biomarkers rather than *ad hoc* molecular lists. Multi-omics work is already linking endometriosis to related conditions such as recurrent implantation failure at the level of shared genes and niche structure. Side-by-side comparison of endometriosis, adenomyosis, and endometriosis-associated ovarian cancer should clarify how much immunology can be borrowed across these entities and where disease-specific biology dominates (22).

Organoid initiatives (e.g., WERF EPHect) and reproductive organ-on-a-chip systems supply more standardized settings to test hypotheses raised by single-cell maps and in silico screens (101). Federated learning and related privacy-preserving schemes offer one way to pool signal across centers without centralizing raw single-cell data—important if models are to train on cohorts that match real-world diversity (102).

Foundation models will probably gain endometriosis-specific fine-tuning; their utility will still depend on task-aware benchmarking, as in other tissues. Linking transcriptomic, epigenomic, proteomic, metabolomic, spatial, and clinical layers in shared analysis workflows is likely to matter as much as any single algorithm class for building testable models of the endometriotic immune niche. In practical terms, AI adds the most value when conventional pipelines struggle with cross-modal integration, high-order interaction patterning, or rank-order prioritization across large candidate spaces; for narrowly scoped annotation tasks, simpler baselines may remain competitive and more interpretable.

A minimal translational pathway can help align expectations: (1) external validation of signatures/targets in independent cohorts with transparent performance reporting; (2) mechanistic confirmation in perturbation-ready systems (organoid, co-culture, and *in vivo* models); and (3) prospective, biomarker-stratified clinical studies that jointly evaluate efficacy, safety, and subgroup transportability.

Reproducibility standards should also be explicit at field level: report accession identifiers and preprocessing choices, provide code/model settings where possible, define benchmark splits and leakage controls, and document cross-center ethical/data-governance procedures for shared or federated analyses.

**Figure 4:**
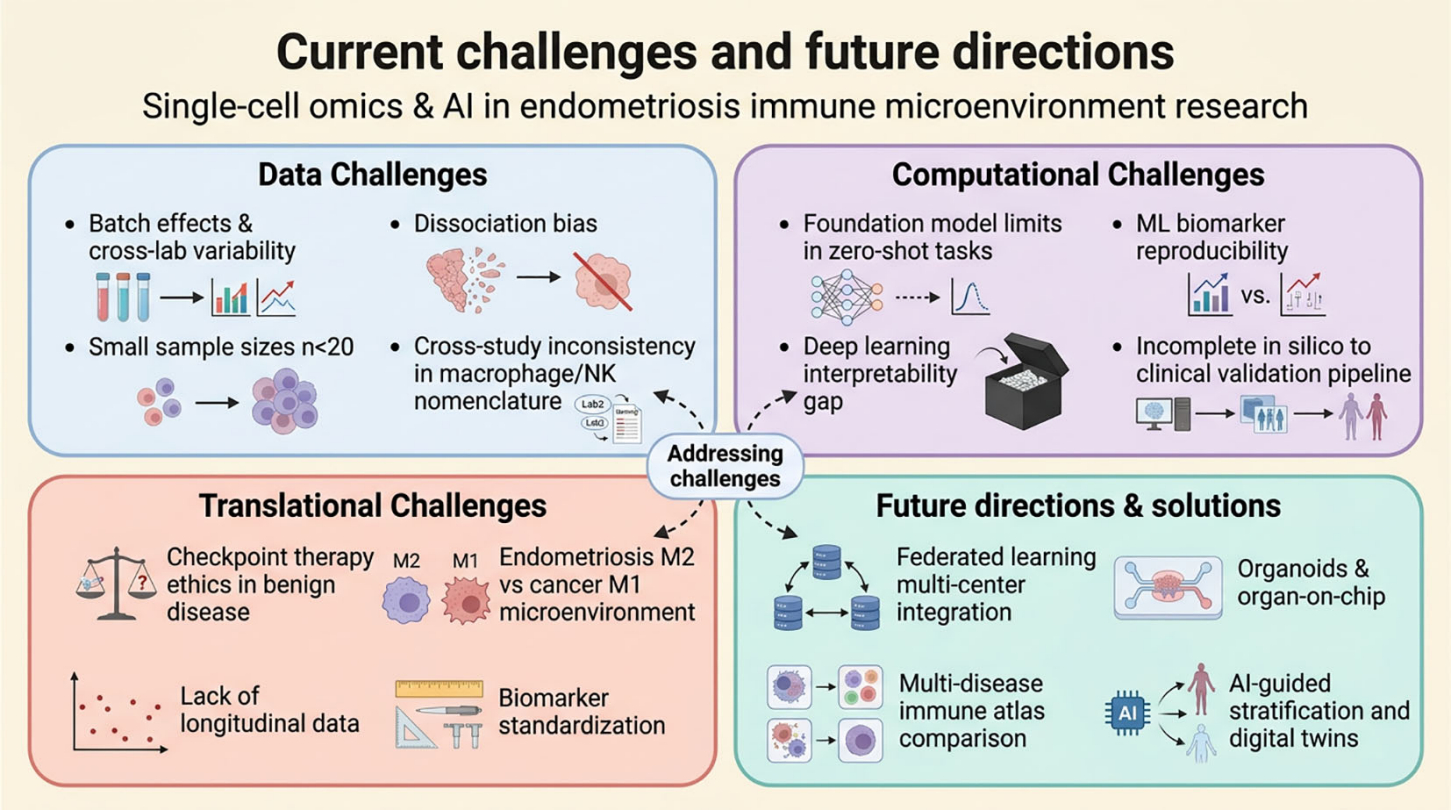
Current challenges and future directions in single-cell omics and AI-driven endometriosis research. Roadmap-style layout. Data: batch effects, dissociation bias, sparse longitudinal sampling. Computation: foundation-model limits on some benchmarks, uneven replication of ML signatures, limited interpretability for deep models. Translation: prediction–experiment gaps, safety/ethics of checkpoint-style therapy in benign disease, need for standardized biomarker workflows. Mitigations under discussion: federated or privacy-preserving integration, organoids and organ-on-chip validation, cross-disease immune comparisons (endometriosis, adenomyosis, ovarian cancer), and trials that use immune stratification when biomarkers are fit for purpose.

Together, single-cell omics and AI are practical tools for refining hypotheses about immune inflammation in endometriosis; converting those hypotheses into durable clinical gains will require the usual mix of replication, prospective validation, and cautious translation, points summarized schematically in **Figure 4**. To move the field forward, future AI and single-cell studies in endometriosis must meet strict reproducibility expectations. First, researchers should publicly share their data (providing clear accessions) and algorithms (code and model settings). Second, the field needs standardized benchmarks to fairly compare different AI models. Finally, when using data from multiple hospitals, strict cross-center ethics must be followed to protect patient privacy. Following these rules will help make AI tools more reliable for clinical use.
